# National Incidence of Intracranial Haemorrhage–Related Hospitalisations and Mortality in England 2014–2019

**DOI:** 10.1155/srat/6671568

**Published:** 2025-09-27

**Authors:** Katherine J. Creeper, Andrew C. Stafford, Allycia MacDonald, Arvind Chandratheva, Alexander T. Cohen

**Affiliations:** ^1^Curtin Medical School, Curtin University, Bentley, Western Australia, Australia; ^2^Haematology Department, PathWest Laboratory Medicine, Nedlands, Western Australia, Australia; ^3^Haematology Department, Sir Charles Gairdner Hospital, Nedlands, Western Australia, Australia; ^4^Neurology Department, Fiona Stanley Hospital, Murdoch, Western Australia, Australia; ^5^Acute Stroke Unit, National Hospital for Neurology and Neurosurgery, London, UK; ^6^Neurology Department, University College London Hospital, London, UK; ^7^Haematology Department, Guy's and St Thomas' NHS Foundation Trust, London, UK

**Keywords:** haemorrhage, hospitalisation, incidence, intracranial, England

## Abstract

**Background:** Intracranial haemorrhage (ICrH) is the most frequent cause of bleeding-related death. However, few studies describe the national incidence of ICrH-related acute hospitalisations and mortality. We report the national burden and incidence of hospitalisation and mortality of ICrH and its subtypes.

**Methods:** A population-based review in England between 2014 and 2019 of acute admissions or deaths was undertaken. Admission and mortality data were obtained from electronic databases (traumatic death data were unavailable). ICrH events were identified by the International Classification of Diseases Version 10 codes. ICrH were subclassified by anatomical site and either traumatic or atraumatic cause.

**Results:** In the 6-year study period, there was a total of 468,996 hospitalisations for ICrH, of which 280,003 (59.7%) were atraumatic and 188,993 (40.3%) were traumatic. Then, 50,004 atraumatic ICrH-related deaths were recorded; of these deaths, 43,061 were subclassified by anatomical site. The mean annual incidence rates (per 100,000 person years) were 141.0 for ICrH-related hospitalisations and 15.0 for atraumatic ICrH-related mortality. Males had a 7% higher incidence rate for atraumatic ICrH-related hospitalisations (OR 1.07, 95% CI 1.05–1.09, *p* < 0.0001). Females had a higher mean annual atraumatic ICrH-related mortality (OR 1.21, 95% CI 1.16–1.26, *p* < 0.0001). Then, 23.4% (*n* = 109,770) of all ICrH hospitalisations occurred in patients ≥ 85 years.

**Conclusion:** The majority of ICrH acute hospitalisations (59.7%) were atraumatic. Sex differences were seen in outcome measurements: males had a higher overall incidence of hospitalisation; however, females had a higher incidence of atraumatic ICrH-related mortality.

## 1. Introduction

Intracranial haemorrhage (ICrH) refers to bleeding within the cranium, including brain parenchyma and surrounding spaces [1, 2]. It encompasses four broad types: extradural, subdural, intracerebral (with or without intraventricular haemorrhage) and subarachnoid. The aetiology, clinical features and prognosis of each type are different. Intracerebral haemorrhage is often nontraumatic bleeding into the brain parenchyma; conversely, subdural and extradural haemorrhages are often traumatic. Subarachnoid haemorrhage may be caused by cerebral aneurysms, arteriovenous malformations, vasculitis and trauma [3].

The most important associated pathologies for ICrH include trauma, hypertensive vascular disease and cerebral amyloid angiopathy. Risk factors include age, sex, smoking, alcohol, hypercholesterolaemia, antiplatelet and anticoagulant use, sympathomimetic drugs, ethnicity, socioeconomic status and genetics [1, 3].

However, data on the incidence of ICrH bleeding-related hospitalisation classified by traumatic versus atraumatic aetiology as well as anatomical subtype are lacking. The aim of this study was therefore to report a more detailed analysis of the national burden and incidence of hospitalisation and mortality of intracranial bleeding in England between 2014 and 2019.

## 2. Methodology

### 2.1. Study Design and Participants

A population-based analysis was conducted covering all individuals in England between 2014 and 2019 who were either admitted to an acute care ward in a National Health Service (NHS) hospital or who died during that period. This included hospital admissions for children. Data on hospitalisations were sourced from the Hospital Episode Statistics (HES) database, a publicly accessible resource that contains clinical information such as admission diagnoses (coded using the International Classification of Diseases, 10th Revision [ICD-10]), along with demographic details like age, sex, administrative records and geographical location [4]. Reporting to HES is mandatory for all NHS hospital admissions across the United Kingdom; however, for this analysis, only data from English NHS hospitals—approximately 700 in total—were considered.

Mortality information was retrieved from the Office for National Statistics (ONS), which compiles death records across the United Kingdom based on official death certificates [5]. These records include data on the year of death, age, sex and the main cause of death, categorised using ICD-10 codes [6, 7]. Deaths due to ICrH, whether they occurred in or out of hospital, were included. However, the time interval between a hospital admission for ICrH and subsequent death was not examined. The mortality data was restricted to residents of England. Since trauma-related ICrH deaths are usually coded by the type of injury rather than the resulting medical condition, the analysis focused only on deaths from nontraumatic causes. Incidence and mortality rates for nontraumatic ICrH were presented together to illustrate their relationship more clearly. These rates were calculated using yearly population estimates provided by the ONS. A summary of the data and its sources is provided in Table 1.

### 2.2. Inclusion Criteria

The HES and ONS databases record the reasons for hospital admission and cause of death, respectively, using the ICD-10 coding system. Previous research has outlined the specific ICD-10 codes associated with ICrH [7]. This analysis included all patients admitted with a diagnosis of ICrH. Data selection was based on ICD-10 classifications, as defined by the International Statistical Classification of Diseases and Related Health Problems (10th edition). A complete list of the codes and categories used in the analysis can be found in Table S1 [7].

### 2.3. Exclusion Criteria

Patients who were treated and discharged directly from an emergency department or outpatient clinic were excluded from the hospitalisation analysis. Additionally, any data falling outside the study period were omitted. In particular, data from the year 2020 were excluded due to possible disruptions and delays in reporting related to the COVID-19 pandemic.

### 2.4. Statistical Analysis

The data were organised using Microsoft Excel (Microsoft Corporation, Redmond, WA, United States) and categorised by year, sex, anatomical location and whether the ICrH was traumatic or nontraumatic. To support the analysis of this large dataset, the 25 relevant ICD-10 codes (160.0–162.9 and S06.4–S06.6) were grouped into four categories, as outlined in Table S1.

Hospital admission and mortality rates were calculated using population estimates from the ONS and reported as rates per 100,000 patient-years and per 100,000 individuals, respectively [9, 10]. Data are expressed as means with standard deviations or as percentages, depending on the variable.

Proportion comparisons were used to calculate relative risks and odds ratios using corrected chi-square tests. Annual odds ratios were determined with the chi-square test for trend, conducted via StatCalc software (Centers for Disease Control and Prevention, Atlanta, GA, United States). A *p* value of less than 0.05 (two-tailed) was considered statistically significant for all analyses [11].

## 3. Results

There was a total of 468,996 hospitalisations for ICrH with a mean of 78,166 ± 6382 (standard deviation) per year (Table 2). Over the 6-year study period, the incidence rate for total ICrH hospitalisations increased (chi-square test for trend *χ*^2^ = 2270, *p* value < 0.0001). Of all ICrH hospitalisations, 280,003 (59.7%) were atraumatic and 188,993 (40.3%) were traumatic. A total of 50,004 atraumatic ICrH-related deaths were recorded in the 6-year study period with a mean of 8334 ± 144 per year. Of these, 43,061 were subclassified according to ICD-10 codes, with 6943 were unclassified. The mean annual incidence rate per 100,000 person years for ICrH-related hospitalisations was 141 and atraumatic-related mortality was 15.

### 3.1. Hospitalisations and Mortality by Sex

There was a total of 256,130 (54.6%) hospitalisations in males and 212,733 (45.4%) in females. Of the 256,130 male ICrH-related hospitalisations, 113,204 (44.2%) were traumatic. Of the 212,733 female ICrH-related hospitalisations, 75,745 (35.6%) were traumatic. Compared to females, males had a 7% higher incidence rate for atraumatic-related ICrH-related hospitalisations (OR 1.07, 95% CI 1.05–1.09, *p* < 0.0001) and a 53% higher incidence rate for traumatic ICrH-related hospitalisations (OR 1.53, 95% CI 1.50–1.57, *p* < 0.0001).

**Figure 1:**
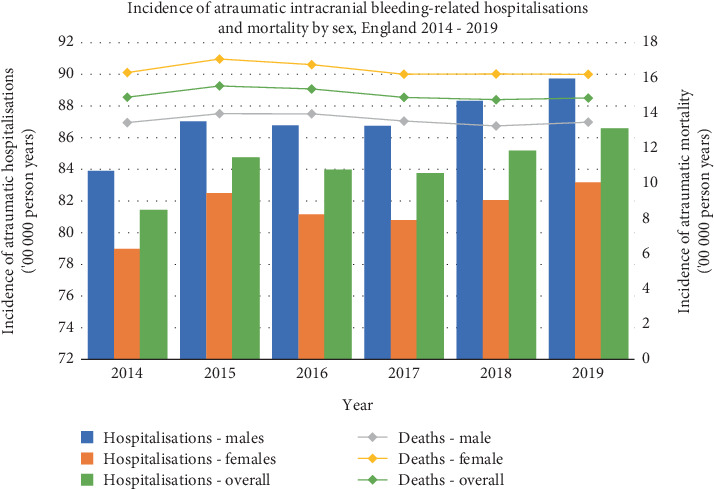
Incidence of atraumatic intracranial haemorrhage–related hospitalisations and mortality by sex, England 2014–2019.

Of the 50,004 atraumatic intracranial bleeding-related deaths, 27,672 (55.3%) occurred in females and 22,332 (44.7%) were in males. Females had a higher mean annual atraumatic-related mortality (OR 1.21, 95% CI 1.16–1.26, *p* < 0.0001) (Figure 1).

There were sex differences in the frequency of ICrH atraumatic-related mortality subtypes. Females had a total atraumatic-related mortality of 6230 (63.6%) subarachnoid haemorrhages compared to males, who had 3554 (36.4%). Males had 2560 (61.3%) subdural haemorrhage atraumatic-related deaths compared to 1621 (38.7%) in females. Then, 15,915 (54.8%) intracerebral haemorrhage–related atraumatic deaths occurred in females and 13,135 (45.2%) in males. Males and females had the same atraumatic-related extradural haemorrhage mortality of 23 (50%).

### 3.2. Hospitalisations and Mortality by Age

**Figure 2:**
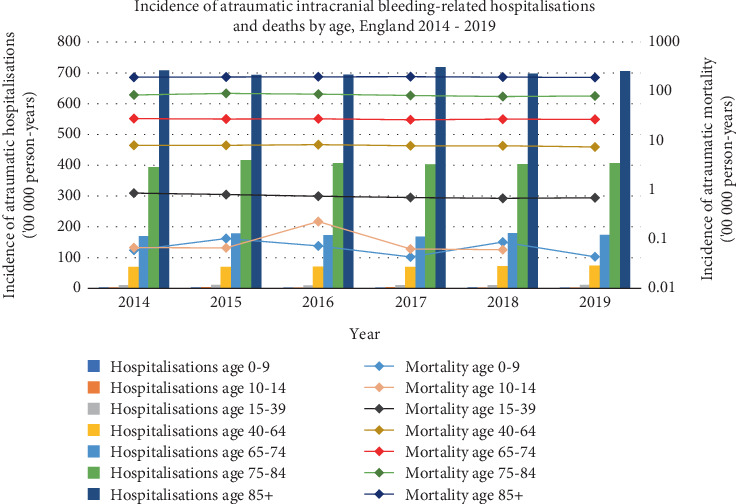
Incidence of atraumatic intracranial haemorrhage–related hospitalisations and deaths by age, England 2014–2019.

Then, 82,094 (17.5%), 125,496 (26.7%) and 109,770 (23.4%) of all ICrH-related hospitalisations occurred in patients 65–74, 75–84 and ≥ 85 years, respectively. Of the 188,993 traumatic ICrH-related hospitalisations, 25,580 (13.5%), 47,980 (25.4%) and 53,011 (28%) occurred in patients 65–74 years, 75–84 and ≥ 85 years, respectively (Figure 2).

Mean incidence rates for all ICrH-related hospitalisations and mortality across the 6-year study period stratified by age are presented in Table 3.

### 3.3. Hospitalisations and Mortality by Anatomical Bleeding Site

Then, 10,594 (2.2%) of all ICrH-related hospitalisations were extradural, 152,024 (32.4%) were intracerebral, 112,386 (24%) were subarachnoid, 180,829 (38.5%) were subdural and 13,163 (2.8%) were nonspecific. All of the 152,024 intracerebral ICrH-related hospitalisations were atraumatic. This is in comparison to 9880 (93.3%) of the total 10,594 extradural ICrH, which were traumatic. Then, 128,433 (71%) of subdural ICrH were atraumatic, and 52,396 (29%) were traumatic.

**Figure 3:**
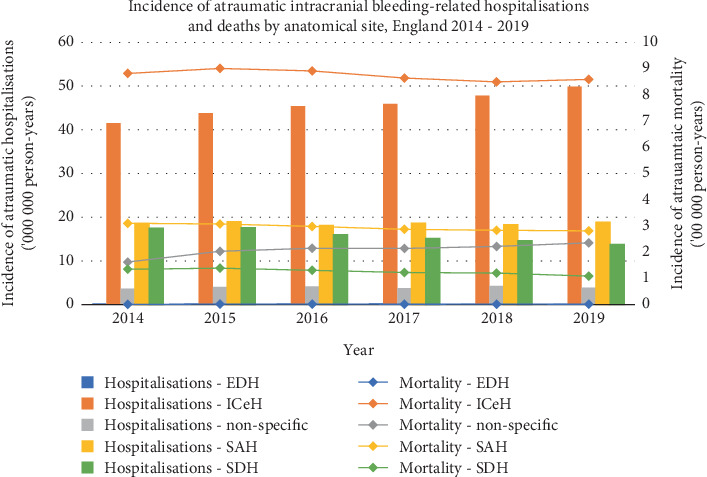
Incidence of atraumatic intracranial haemorrhage–related hospitalisations and deaths by anatomical site, England 2014–2019. EDH, extradural; SAH, subarachnoid; ICeH, intracerebral; SDH, subdural.

Of the 50,004 atraumatic ICrH-related deaths, 46 (0.09%) were extradural, 29,050 (58%) were intracerebral, 9784 (19.6%) were subarachnoid, 4181 (8.4%) were subdural and 6943 (13.8%) were not classified (Table 4 and Figure 3).

## 4. Discussion

This study provides contemporary insight into the national incidence of hospitalisations and associated atraumatic mortality due to ICrH in England. There was a total of 468,996 ICrH-related hospitalisations during the study period with a mean of 78,166 ± 6382 per year. Of these, 188,993 (40.3%) were traumatic and 280,003 (59.7%) were atraumatic. A total of 50,004 atraumatic ICrH-related deaths were recorded in the study period with a mean of 8334 ± 144 per year. Males had a higher mean annual incidence of hospitalisation due to ICrH. Females had a higher annual incidence of atraumatic bleeding-related mortality (OR 1.21, 95% CI 1.16–1.26, *p* < 0.00001). Then, 23.4% (*n* = 109,770) of all ICrH-related hospitalisations occurred in patients ≥ 85 years. Of the 188,993 traumatic ICrH-related hospitalisations, 28% (*n* = 53,011) occurred in patients ≥ 85 years. Then, 180,829 (38.5%) of ICrH-related hospitalisations were subdural. Then, 29,050 (58%) of atraumatic ICrH-related deaths were intracerebral.

We observed a trend towards increasing incidence of both traumatic and atraumatic ICrH-related hospitalisations across the 6-year study period; however, the mortality rate remained stable. It is hypothesised that the observation of increasing incidence of ICrH-related hospitalisations is not only due to the ageing population but also the increased occurrence of risk factors for stroke and intracerebral haemorrhage such as obesity, chronic kidney disease, elevated blood sugar, sedentary lifestyle and excessive alcohol consumption [12, 13]. Interestingly, studies have documented that treatment of chronic hypertension, a well-documented risk factor for intracerebral haemorrhage, improved during the same time period [12, 14, 15]. It could also be speculated that the increased use of anticoagulants for stroke prophylaxis in atrial fibrillation and the expected accompanying increase in hospitalisations and mortality was counterbalanced by the switch from vitamin K antagonists to direct oral anticoagulants (Xa inhibitors). We hypothesise that the mortality rate remained stable despite increasing hospitalisation due to improved patient care, better management of comorbidities and advanced reversal strategies for anticoagulation.

Over the 6-year study period, the use of direct oral factor Xa inhibitors (DOXIs) increased [15]. The use of concurrent medications, including anticoagulation and/or antiplatelet treatment for our cohort, is unknown. Despite the improved safety profile and association with reduced rates of ICrH of DOXIs compared to vitamin K antagonists, we observed little variability in the rates of atraumatic ICrH-related mortality between 2014 and 2019. This may be due to improvements in critical care for those with ICrH, as well as an increased willingness of clinicians to prescribe anticoagulation, thus potentially offsetting any potential advantage [13].

Similarly, we also identified an increased absolute incidence of ICrH hospitalisation yet a lower incidence of ICrH-related atraumatic mortality in males compared to females. Possible explanations for the increased incidence of ICrH hospitalisation in males may include a higher prevalence of vascular risk factors such as hypertension, smoking and diabetes compared to females. In contrast, the increased incidence of ICrH-related atraumatic mortality in females may be due to a higher incidence of subarachnoid haemorrhage and intracerebral haemorrhage, worse baseline level of health, more advanced age and greater severity of haemorrhage than in males [17, 18]. Multiple studies have also demonstrated that women have an increased prevalence of atrial fibrillation and therefore potentially greater usage of anticoagulation and/or antiplatelet therapy, which may partially account for our results [18, 19].

The reported mortality rate of 50,004 (17.8%) intracerebral haemorrhages for a total of 280,000 atraumatic hospitalisations is in line or possibly slightly lower than previously reported studies [20, 21]. It is well recognised that the mortality rate following ICrH is highest in the first 30 days and then plateaus [20]. Early death after an ICrH event is often attributable to the ICrH itself and is increased by concurrent oral anticoagulant use, advanced age and uncontrolled hypertension [20, 22]. We hypothesise that our mortality rate may be slightly lower than expected or previously reported in the literature due to the knowledge that many of the deaths occur as a result of traumatic intracerebral haemorrhage; this data, however, is not available for inclusion within our review. Another possible explanation is that our data included significant numbers of subdural and epidural haemorrhages, and these have a lower mortality rate.

The high incidence of subdural haemorrhage–related hospitalisations may be due to the both acute and chronic presentations being coded as one entity. Their low incidence of mortality, however, likely reflects that these haemorrhages are frequently due to traumatic injuries [3]. In contrast, subarachnoid haemorrhage can be due to trauma, cerebral aneurysms, arteriovenous malformations or underlying connective tissue disease. Deaths secondary to trauma are reported to the coroner and are reported by the external cause (i.e., assault by blunt or sharp object).

This study offers significant benefits, allowing for the visualisation of longitudinal trends in ICrH-related hospitalisations and mortality rates within prospective, population-based data. Stratification by sex, age and anatomical classification enabled diverse analyses, unveiling various facets of these hospitalisations and mortality patterns. By restricting the analysis to pre-2020 data, the study mitigates any influence stemming from altered resource allocation or changes in care due to the COVID-19 global health crisis [23].

## 5. Limitations

Limitations include the potential inaccuracy in database recordings, which is common in large datasets where multiple individuals contribute to data classification and entry. While the HES database is increasingly utilised for research and quality improvement, concerns about data accuracy have been raised. For example, there were 44 subjects that did not have an assigned sex. Efforts are underway by relevant medical bodies to address these issues [24].

Traumatic presentations of ICrH are subclassified by ICD as SO6.4 (epidural), SO6.5 (subdural) and SO6.6 (subarachnoid), as outlined in the Table S1. Unfortunately, data on traumatic intracerebral haemorrhage are not publicly available and may account for the lower mortality rate reported in our manuscript. There is also the possibility that the type of ICrH may have been incorrectly classified, including both anatomical site and also the distinction as traumatic versus atraumatic.

Third, we recognise that it is not unusual for patients with ICrH to have a high readmission rate, and as a result, the same patient may potentially may have been included on more than one occasion. This may be offset by the underreporting of ICrH incidence. This is because those patients had ICrHs not requiring admission to hospital, or those that had catastrophic haemorrhages who died prior to reaching hospital (for example) were not included in this analysis.

Fourth, the calculation of incidence rates based on population estimates derived from the census involves assumptions about migration patterns, birth and death trends. While the accuracy of these estimates was deemed acceptable in a 2021 review, they remain approximations that could influence the study's results, albeit to a limited extent [25].

Moreover, there are concerns regarding coding bias and the absence of consideration for various risk factors and confounding variables. Both the HES and ONS databases lack patient-level data, which precludes access to information on concurrent medications, comorbidities like hypertension, cancer or renal failure, as well as demographic details on race and socioeconomic status. Knowledge of these factors could have elucidated trends in bleeding risk across age, sex or anatomical sites. Geographical knowledge may have also provided insight into access to primary care for risk factor management as well as acute management for stroke.

## 6. Conclusion

Publicly available data allow large, retrospective epidemiological analysis. To date, the data on the incidence of ICrH and atraumatic-related mortality have been limited. This is the first published report documenting the national incidence of hospitalisations and atraumatic associated mortality in England. The observed increase in hospitalisations highlights the importance of continued efforts to prevent and treat modifiable risk factors as well as the continuation of strategies designed to streamline acute care pathways in an attempt to address mortality.

## Figures and Tables

**Table 1:** Overview detailing sites of data collated.

	**HES database**	**ONS database**
Main diagnosis by ICD-10 code	√	√
All diagnoses by ICD-10 code	√	
Age	√	√
Sex	√	v
Annual population estimates		√
Annual reporting cycle	April–March (following year)	January–December

Abbreviations: HES, Hospital Episode Statistics; ICD-10, International Classification of Disease Version 10; ONS, Office for National Statistics.

**Table 2:** Summary of overall results.

	**Total ICrH-related hospitalisations**	**Traumatic ICrH-related hospitalisations**	**Atraumatic ICrH-related hospitalisations**	**Total atraumatic ICrH-related mortality**
Total (*n*, % of total)	468,996	188,993 (40.3)	280,003 (59.7)	50,004
Mean per year ± std dev	78,166 ± 6381.7	31,498.8 ± 5085.6	46,667.2 ± 1373.6	8,334 ± 144
Mean annual incidence rate per 100,000 person years^a^	141	56.8	84.2	15

^a^Crude person years based on population figures.

**Table 3:** Incidence rates for total hospitalisation and atraumatic-related mortality between 2014 and 2019 stratified by age.

	**Age group (years)**						
	**0–9**	**10–14**	**15–39**	**40–64**	**65–74**	**75–84**	**85+**
Mean incidence rate of hospitalisations (standard deviation)	12.6 (1.0)	9.2 (0.9)	30.3 (1.7)	103.6 (6.1)	252.4 (16.0)	652.6 (40.0)	1357.1 (132.3)
Mean incidence rate mortality (standard deviation)	0.07 (0.02)	0.08 (0.08)	0.75 (0.07)	7.92 (0.28)	27.33 (0.54)	84.17 (4.63)	195.33 (2.68)

*Note:* Data is per 100,000 person years.

**Table 4:** Total number of hospitalisations classified by anatomical site and traumatic vs. atraumatic associated presentations.

	**Extradural**	**Subdural**	**Intracerebral**	**Subarachnoid**	**Not classified**	**Total**
Atraumatic	714	52,396	152,024	61,706	13,163	280,003
Traumatic	9880	128,433	0	50,680	0	188,993
Total	10,594	180,829	152,024	112,386	13,163	468,996

## Data Availability

The data that support the findings of this study are available from the corresponding author upon reasonable request.

## Supporting Information

Supporting Information Additional supporting information can be found online in the Supporting Information section. Table S1 includes the full list of included ICD-10 codes and anatomical and traumatic versus atraumatic classifications that were used for analysis.
