# Supplementary material for: National Incidence of Intracranial Haemorrhage–Related Hospitalisations and Mortality in England 2014–2019
Source: Stroke Res Treat. 2025 Sep 27;2025:6671568. doi: 10.1155/srat/6671568 (PMC12496138; doi:10.1155/srat/6671568)
Supplement: Supporting Information — Additional supporting information can be found online in the Supporting Information section. Table S1 includes the full list of included ICD-10 codes and anatomical and traumatic versus atraumatic classifications that were used for analysis. [file 6671568.f1.docx]

**Supplementary Table 1: ICD-10 codes and classifications included in analysis.**

| **ICD-10 Code** | **Description** | **Anatomical classification** | **Traumatic or atraumatic** |
| --- | --- | --- | --- |
| I60.0 | Subarachnoid haemorrhage from carotid siphon and bifurcation | Subarachnoid | Atraumatic |
| I60.1 | Subarachnoid haemorrhage from middle cerebral artery | Subarachnoid | Atraumatic |
| I60.2 | Subarachnoid haemorrhage from anterior communicating artery | Subarachnoid | Atraumatic |
| I60.3 | Subarachnoid haemorrhage from posterior communicating artery | Subarachnoid | Atraumatic |
| I60.4 | Subarachnoid haemorrhage from basilar artery | Subarachnoid | Atraumatic |
| I60.5 | Subarachnoid haemorrhage from vertebral artery | Subarachnoid | Atraumatic |
| I60.6 | Subarachnoid haemorrhage from other intracranial arteries | Subarachnoid | Atraumatic |
| I60.7 | Subarachnoid haemorrhage from intracranial artery, unspecified | Subarachnoid | Atraumatic |
| I60.8 | Other subarachnoid haemorrhage | Subarachnoid | Atraumatic |
| I60.9 | Subarachnoid haemorrhage, unspecified | Subarachnoid | Atraumatic |
| I61.0 | Intracerebral haemorrhage in hemisphere, subcortical | Intracerebral | Atraumatic |
| I61.1 | Intracerebral haemorrhage in hemisphere, cortical | Intracerebral | Atraumatic |
| I61.2 | Intracerebral haemorrhage in hemisphere, unspecified | Intracerebral | Atraumatic |
| I61.3 | Intracerebral haemorrhage in brain stem | Intracerebral | Atraumatic |
| I61.4 | Intracerebral haemorrhage in cerebellum | Intracerebral | Atraumatic |
| I61.5 | Intracerebral haemorrhage, intraventricular | Intracerebral | Atraumatic |
| I61.6 | Intracerebral haemorrhage, multiple localized | Intracerebral | Atraumatic |
| I61.8 | Other intracerebral haemorrhage | Intracerebral | Atraumatic |
| I61.9 | Intracerebral haemorrhage, unspecified | Intracerebral | Atraumatic |
| I62.0 | Subdural haemorrhage (acute)(nontraumatic) | Subdural | Atraumatic |
| I62.1 | Nontraumatic extradural haemorrhage | Extradural | Atraumatic |
| I62.9 | Intracranial haemorrhage (nontraumatic), unspecified | Non-specific | Atraumatic |
| S06.4 | Epidural haemorrhage | Extradural | Traumatic |
| S06.5 | Traumatic subdural haemorrhage | Subdural | Traumatic |
| S06.6 | Traumatic subarachnoid haemorrhage | Subarachnoid | Traumatic |
